# Measuring and Enhancing the Ionic Conductivity of Chloroaluminate Electrolytes for Al-Ion Batteries

**DOI:** 10.1021/acs.jpcc.3c02302

**Published:** 2023-07-06

**Authors:** Anthony J. Lucio, Iwan Sumarlan, Elena Bulmer, Igor Efimov, Stephen Viles, A. Robert Hillman, Christopher J. Zaleski, Karl S. Ryder

**Affiliations:** † Centre for Sustainable Materials Processing, School of Chemistry, University of Leicester, Leicester LE1 7RH, U.K.; ‡ Department of Chemistry, University of Mataram, Jl. Majapahit. No. 62, Mataram, 83115 Lombok, Indonesia; § Department of Chemical and Biological Engineering, University of Sheffield, Sheffield S1 3JD, U.K.; ∥ Biotechnology Group, School of Chemistry, University of Leicester, Leicester LE1 7RH, U.K.

## Abstract

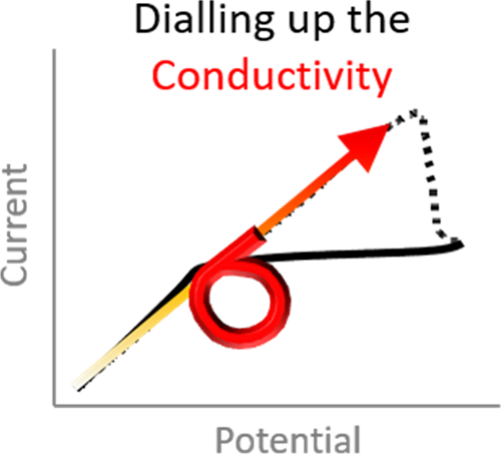

At the core of the aluminum (Al) ion battery is the liquid electrolyte, which governs the underlying chemistry. Optimizing the rheological properties of the electrolyte is critical to advance the state of the art. In the present work, the chloroaluminate electrolyte is made by reacting AlCl_3_ with a recently reported acetamidinium chloride (Acet-Cl) salt in an effort to make a more performant liquid electrolyte. Using AlCl_3_:Acet-Cl as a model electrolyte, we build on our previous work, which established a new method for extracting the ionic conductivity from fitting voltammetric data, and in this contribution, we validate the method across a range of measurement parameters in addition to highlighting the model electrolytes’ conductivity relative to current chloroaluminate liquids. Specifically, our method allows the extraction of both the ionic conductivity and voltammetric data from a single, simple, and routine measurement. To bring these results in the context of current methods, we compare our results to two independent standard conductivity measurement techniques. Several different measurement parameters (potential scan rate, potential excursion, temperature, and composition) are examined. We find that our novel method can resolve similar trends in conductivity to conventional methods, but typically, the values are a factor of two higher. The values from our method, on the other hand, agree closely with literature values reported elsewhere. Importantly, having now established the approach for our new method, we discuss the conductivity of AlCl_3_:Acet-Cl-based formulations. These electrolytes provide a significant improvement (5–10× higher) over electrolytes made from similar Lewis base salts (e.g., urea or acetamide). The Lewis base salt precursors have a low economic cost compared to state-of-the-art imidazolium-based salts and are non-toxic, which is advantageous for scale-up. Overall, this is a noteworthy step at designing cost-effective and performant liquid electrolytes for Al-ion battery applications.

## Introduction

The need for alternative energy storage, transmission, and conversion technologies is urgent in order to meet growing global demands. Renewable energy sources, such as wind and solar, are playing a vital role in the move toward net zero targets.^1−3^ However, due to the intermittent nature of these renewable sources, there is a real need for competitive, stationary (grid-scale) energy storage options. Lithium (Li)-ion battery technology represents the most mature and widespread form of electrochemical energy storage with overwhelming adoption in portable electronic devices. The uneven distribution of critical raw materials and the high economic costs, however, significantly reduce the appeal of Li-ion batteries for large-scale stationary storage.^4−6^ Aluminum (Al)-ion-based batteries are a promising alternative due to the large and wide abundance of Al; they are often made from inexpensive non-toxic materials, and in theory, they can provide competitive energy densities (e.g., 13.4 W h cm^–3^ for Al versus 6.2 W h cm^–3^ for Li).^7^ As a result, non-aqueous Al battery chemistries are receiving significant interest from the scientific community.^8−13^

At the heart of the Al-ion battery is the liquid electrolyte. These electrolytes, often called chloroaluminate liquids, are formed by an acid–base reaction between a Lewis acid (LA; i.e., AlCl_3_ salt) and a Lewis base (LB; e.g., Cl^–^-containing salt). To date, the majority of research has been done on chloroaluminate liquids made from the solid (at room temperature) salt 1-ethyl-3-methylimidazolium chloride (EMIM-Cl);^14−21^ this electrolyte is commercially available.^22^ The electrochemical and rheological performance is promising, but it comes with a high economic cost of the starting material, which ultimately is not advantageous for scale-up. Other liquid electrolytes, often called ionic liquid analogues (ILAs), can be formed by reaction with AlCl_3_ and softer Lewis bases (e.g., urea^23−28^ or acetamide^29−34^). These are much more cost-effective options, but they are limited by their electrochemical and rheological performance.

We have recently reported the formation of chloroaluminate liquids from amidine-based (chloride) salts that exhibit encouraging electrochemical and rheological performance at a significantly lower economic cost.^35^ These liquids are able to accommodate higher concentrations of Al species and represent a credible alternative to the aforementioned Lewis base salts. Lastly, there are also ongoing efforts to develop polymer gelled electrolytes to improve ionic conductivity and address other issues surrounding non-aqueous battery chemistries (i.e., reduce moisture sensitivity, minimize leakage, and allow for the generation of flexible but tough materials).^36,37^ All of this work is aimed at developing performant electrolytes for Al-ion batteries.

The mole ratio of the LA and LB has a significant impact on not only the rheological properties of the electrolyte but also its ability to form a stable liquid. The stable compositional range depends on the LB salt. Specifically, at mole LA:LB ratios <1, the liquids are termed Lewis basic; at LA:LB ratios = 1, the liquid is termed neutral; and at LA:LB ratios >1, the liquids are termed Lewis acidic.^38,39^ Consequently, there is often an ideal mole ratio between the LA and LB that seeks to increase the conductivity (>5 mS cm^–1^) and decrease the viscosity (<50 cP). The Lewis acidity also impacts the electroactive Al species present. Previous literature suggests that Lewis acidic formulations (>1:1) are needed in order for Al deposition to proceed,^27,28,34,39−43^ where there is a mixture of both anionic (e.g., AlCl_4_^–^ and Al_2_Cl_7_^–^) and cationic (e.g., [AlCl_2_(LB)_*n*_]^+^) species present. For example, EMIM-Cl based liquids can be formed within a mole ratio ranging from 0.8:1 to 2:1 (LA:LB).^44^ Zhu et al. reported conductivities increasing from 15 to 17.5 mS cm^–1^ for AlCl_3_:EMIM-Cl liquid for mole ratios between 1.4:1 and 1.7:1 (LA:LB).^45^ Conversely, Azimi et al. reported conductivities decreasing from 22.6 to 14.7 mS cm^–1^ for LA:LB compositions between 1:1 and 2:1 with AlCl_3_:EMIM-Cl liquids.^14^ Other reports have found conductivities of 16.1,^46^ 15.5,^47^ and 9.2 mS cm^–1^^48^ for AlCl_3_:EMIM-Cl liquids with a 1.5:1 mole ratio. On the other hand, for urea-based liquids, the conductivities are an order of magnitude lower than that of EMIM-Cl based liquids. The Azimi group report conductivities increasing from 0.91 (LA:LB = 1.1:1) to 1.45 mS cm^–1^ (LA:LB = 1.3:1) and then decreasing to 1.27 mS cm^–1^ (LA:LB = 1.5:1) for AlCl_3_:urea liquids.^23^ Alternatively, Angell et al. found an increasing conductivity from 1.02 (LA:LB = 1:1) to 1.17 mS cm^–1^ (LA:LB = 1.5:1) for AlCl_3_:urea liquids.^25^ It is apparent that there are several factors that can impact the conductivity, such as using different measurement methods, varying purities of LA/LB salts, and temperature. There is a significant need to improve the conductivity to enhance the performance of the liquid electrolyte, and this point has been reiterated by several groups.^14,25,40,49−51^

Conductivity (σ) is an important rheological metric, and increasing the ionic conductivity for chloroaluminate electrolytes is an important step toward advancing Al-ion battery chemistries. Conductivity is conventionally measured using commercially available sensors via an impedance measurement. These often consist of two to four electrodes arranged in a fixed position relative to one another. An AC voltage (single-frequency impedance measurement) is applied to the electrodes that generates a current response in the medium, and the corresponding ohmic resistance (*R*) is used to calculate a conductivity value. The cell constant (*K*) of the sensor, which is dependent on the geometrical arrangement and surface area of the electrodes, governs this calculation. Commercial sensors are often equipped with a thermocouple to accurately measure the solution temperature as conductivity is a temperature-dependent parameter. A more formal method to measure the conductivity can be achieved using frequency response analysis. Here, a broadband impedance spectrum is collected across a range of frequencies and subsequently modeled using an appropriate electrochemical equivalent circuit to extract the resistance value. This method requires that the experimental cell is previously calibrated (ideally against standard conductivity solutions) to find the cell constant. With the resistance and cell constant values, it is straightforward to calculate the conductivity via σ = *K*/*R*. An obvious benefit to this method is a cost-effective, bespoke measurement cell that can be readily produced from common laboratory materials. Lastly, previous work has demonstrated that the conductivity can even be extracted from pulsed voltammetric potentiostatic techniques.^52^

The work presented herein is a detailed exploration into a novel method of measuring ionic conductivity via voltammetric data for ILAs made from the LA salt AlCl_3_ and the LB salt acetamidinium chloride (Acet-Cl). In our earlier work, we established the concept and theory.^35^ We have now taken this one step further, and in the present work, our method is benchmarked to standard AC impedance conductivity measurements. We explore several perturbations on the voltammetric measurement in an effort to optimize individual parameters, and we compare our novel method to a range of other state-of-the-art ILAs. The voltammetric method yields conductivity values of the same order of magnitude (although a factor of two larger) as standard AC impedance techniques and follows similar conductivity trends. Therefore, this method allows for the estimation of conductivity without the need for an additional measurement or sensor. Another aspect of this work is aimed at enhancing the conductivity of electrolyte formulations for Al-ion batteries. With our AlCl_3_:Acet-Cl formulations, we find substantial improvements (5–10× higher ionic conductivity) over analogous liquids made from similar LB salts. Overall, the AlCl_3_:Acet-Cl electrolyte represents a significant step toward improving electrolyte materials for Al-ion battery systems.

## Experimental Section

### Chemicals

The solid Lewis base (LB) salts acetamidinium chloride (Acet-Cl; >98%, Acros-Organics), acetamide (Acet; 99%, Alfa Aesar), urea (ReagentPlus, ≥99.5%, pellets, Sigma-Aldrich), and 1-ethyl-3-methylimidazolium chloride (EMIM-Cl; ≥95%, Aldrich) were used as received. The solid Lewis acid (LA) salt aluminium chloride (AlCl_3_; anhydrous, granular, 99%, Alfa Aesar) was used as received. Paraffin oil (puriss., meets analytical specification of Ph. Eur., BP, viscous liquid, Sigma-Aldrich) was used as received. Conductivity standards (certified traceable to NIST, VWR) of 44,479, 11,419, 8863, 1249, 885, 442, and 74 μS cm^–1^ (at 19 °C) were used as received.

### Electrolyte Synthesis

The ILA electrolytes were prepared by mixing the desired Lewis base salt and Lewis acid salt, at a specified molar ratio, with heating under a protective hydrocarbon layer until all AlCl_3_ dissolved, as demonstrated previously.^28,35^ The liquid was allowed to stir at 50 °C for 1 h before use. This synthesis method produced stable, dark brown liquid electrolytes for electrochemical testing.

### Simultaneous Cyclic Voltammetry (CV) and Quartz Crystal Microbalance (QCM) Measurements

The electrochemical CV/QCM measurements were done with a Reference600 potentiostat (Gamry) coupled to an eQCM10M resonator (Gamry), which allows for simultaneous collection of voltammetric and acoustic signals. The working electrode was a 9.00 MHz (±30 kHz) AT-cut quartz crystal resonator (Seiko) with Pt (electrolyte facing) and Pt (air facing)-coated sides. The Pt surfaces are sputtered to a thickness of 300 nm and polished to a mirror finish with a surface roughness of approximately 60 nm. The total surface area of the Pt working electrode is 0.236 cm^2^, but the exposed surface area was controlled with the cell geometry to be approximately 0.196 cm^2^. The counter electrode was a coiled 2.0 mm dia. Al wire (99.9998%, metals basis, Alfa Aesar) that had a surface area 50*x* that of the working electrode. The quasi reference electrode (QRE) was a straight, bare 2.0 mm dia. Al wire (99.9998 %, metals basis, Alfa Aesar) that provided reproducible potentials. The counter electrode and QRE were positioned 1.5 and 1 cm away from the working electrode, respectively. The CV/QCM cell was made in-house to physically fit the resonator electrodes (refer to ESI 1, Figure S1a, b). A PEEK material was used to provide both chemical inertness against the electrolytes examined and robustness for positioning. All CV/QCM measurements were done directly after EIS measurements at a temperature of 23 ± 3 °C. The measurement solution was quiescent.

For temperature-dependent CV/QCM measurements, the working electrode was a 10 MHz AT-cut polished shear-mode quartz crystal resonator (ICM Manufacturing) with Pt (electrolyte facing) and Au (air facing)-coated sides. The total surface area of the Pt working electrode is 0.21 cm^2^, but the exposed surface area was controlled with the cell geometry to be approximately 0.153 cm^2^. The counter electrode was an Al flag (surface area = 2.34 cm^2^), and a straight, bare 2.0 mm dia. Al wire was used as a QRE. The counter electrode and QRE were positioned 3.5 and 1 cm away from the working electrode, respectively. The temperature-dependent CV/QCM cell was made in-house using a jacketed glass cell with a PTFE cap that provided reproducible positioning. The temperature was varied from 25 to 70 °C using a heated water bath. The measurement solution was quiescent.

### Electrochemical Impedance Spectroscopy (EIS) Measurements

The EIS measurements were done using a COMPACTSTAT.h mobile electrochemistry potentiostat (Ivium). A two-electrode setup was employed in a bespoke jacketed glass cell and a Teflon cap with ports for the two electrodes and gas purging (refer to ESI 1, Figure S1c). Straight, identical length Al wires of 2.0 mm diameter (99.9998%, metals basis, Alfa Aesar) were used for the electrodes. The EIS conductivity cell was calibrated using seven conductivity standards to obtain the cell constant, *K*, 0.377 (±0.002) cm^–1^ that was used to convert measured resistance (*R*; Ω) values from ILAs into conductivity (σ; S cm^–1^) values using the equation σ = *K*/*R* (refer to ESI 2, Figure S2). All EIS measurements were performed directly after synthesis at a temperature of 27 ± 2 °C. Approximately 35 mL of the ILA was pipetted into the glass cell underneath a protective paraffin oil layer. The measurement solution was quiescent. The broadband EIS data were collected at 0 V within a frequency range of 100,000 and 10 Hz with 15 points per decade and an AC voltage amplitude of 10 mV. Complex impedance (Nyquist) data were analyzed by fitting to an electrochemical equivalent circuit consisting of a resistor in series with a constant phase element (i.e., *R*-CPE). A traditional minimization objective function, complex nonlinear least squares method, was used to perform the data fitting.^53^

### Commercial Conductivity Probe

A commercial chemical-resistant type conductivity probe (SC72SN-31-AA; Yokogawa) and meter (SC72 Personal Conductivity Meter; Yokogawa) were used to compare to values from the voltammetric- and impedance-based methods. The cell constant for the commercial conductivity probe was 5.2 ± 0.5 cm^–1^, and it has an integrated temperature sensor (accuracy within ±0.7 °C). Measurements were taken at 25 ± 2 °C.

## Results and Discussion

The following sections outline the Al deposition/dissolution chemistry and our novel voltammetric-based conductivity measurement method in detail before examining the effects of experimental and environmental variables, including different potential scan rates, the width of potential excursion, voltammetric cycling, *iR* compensation effects, temperature, and compositional dependencies. The AlCl_3_:Acet-Cl electrolyte was selected as the main candidate for this study in an effort to not only benchmark against more traditional conductivity measurement methods but also to highlight its physiochemical properties compared to other ILAs. A LA:LB mole ratio of 2:1 was used in our tests as it demonstrated the best electrochemical and rheological performance from our compositional work (discussed below). The final section explores the applicability of the voltammetric-based conductivity method to measure different ILA electrolytes with comparisons to literature references.

### Al Deposition/Dissolution

**Figure 1:**
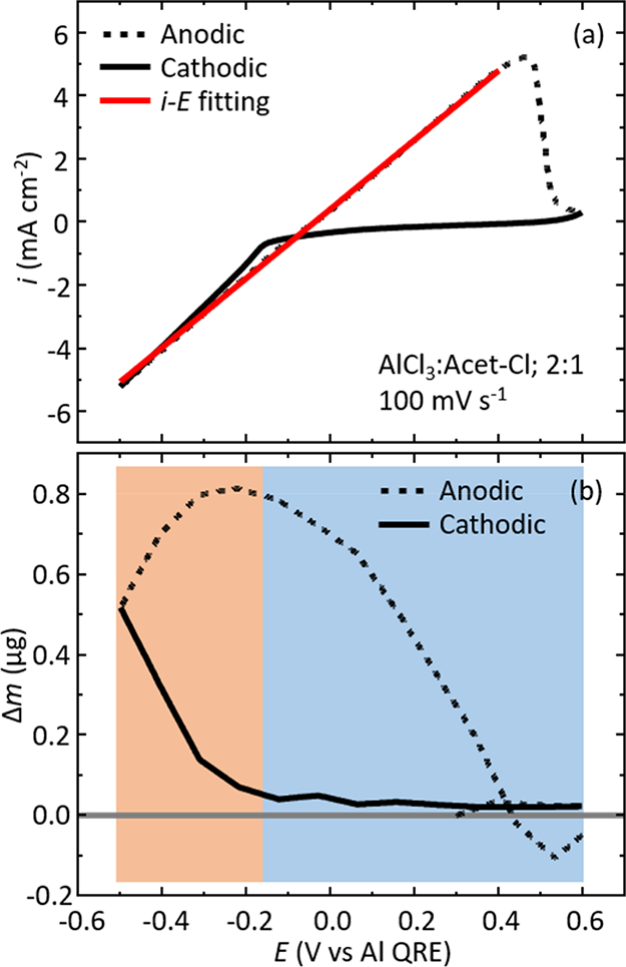
(a) Exemplar experimental cyclic voltammogram (solid and dashed black traces represent cathodic and anodic scans, respectively) demonstrating the *i*–*E* curve-fitting (solid red line) method with the AlCl_3_:Acet-Cl 2:1 electrolyte at a scan rate of 100 mV s^–1^. (b) Corresponding QCM plot at 100 mV s^–1^ showing the mass change as a function of potential. The orange shaded region visually represents deposition potentials, whereas the blue shaded region represents dissolution potentials. A solid gray line is included at the zero mass mark for clarity.

The experimental cyclic voltammogram in Figure 1a shows the response associated with Al deposition and dissolution from the AlCl_3_:Acet-Cl 2:1 formulation on a Pt working electrode at 100 mV s^–1^. The cathodic scan direction is denoted with a solid black line, and the anodic scan direction is a dashed black line. In the cathodic potential scan direction from +0.6 V (vs Al QRE; unless stated otherwise, all potentials are referenced to an Al quasi-reference electrode) to −0.15 V, there are only small (ca. 95 μA cm^–2^) currents that are insignificant on the scale of the redox process being probed. At −0.15 V, the current begins to increase sharply due to the electrochemical reduction of Al^3+^ and consequent deposition of the Al metal on the working electrode. This response continues until the selected vertex potential of −0.5 V. The current signal then shows a very characteristic, linear response in the anodic potential scan direction from −0.5 V to just over +0.4 V, where electrochemical deposition of Al occurs at all potentials negative of −0.15 V and electrochemical dissolution of Al takes place at all potentials positive of −0.15 V. This type of linear voltammetric behavior has been reported previously for similar chloroaluminate liquids.^20,24,34,35,41,42,54,55^ Finally, at +0.5 V, the dissolution current peaks and then falls abruptly as it goes to +0.6 V (the second switching potential), indicating that the deposited Al has been removed.

This redox cycle can also be viewed from electrochemical QCM data obtained concomitantly with the voltammetric data. Figure 1b shows a plot of the mass change (Δ*m*) as a function of potential (*E*) with the solid black line representing the cathodic scan andthe dashed black lines corresponding to the anodic scan. Here, we can see that the scan starts at +0.3 V at the baseline (labeled with the solid gray line) and the potential is swept in the anodic direction to +0.6 V where it increases slightly over the baseline. In the cathodic direction from +0.6 to −0.15 V, there is a slight increase in the mass (due to non-Faradic processes such as potential-dependent adsorption), but beyond −0.15 V, the mass increases significantly as a result of Al deposition on the Pt resonator electrode. The negative vertex potential is reached at −0.5 V, but the mass still increases as the anodic scan direction commences since the current is still negative of the onset deposition potential for Al. The mass increase peaks at 0.8 μg near −0.2 V, indicating a switch from Al deposition to Al dissolution. As the anodic scan continues, the mass decreases and drops below 0 μg. This hysteresis indicates that slightly more mass is lost in the dissolution phase than was deposited in the deposition; however, such responses are often found in the QCM data of similar liquids and may be attributed to the presence of adsorbed species on the Al coating or on the crystal. Taken together, the CV/QCM data indicate that the Al (deposition/dissolution) redox cycle is behaving in a chemically reversible manner.

### Voltammetric-Based Conductivity Measurement

The characteristic linear voltammetric response we see in the anodic potential scan direction can be fit to extract the conductivity. The *i*–*E* curve-fitting method was first established in our previous work,^35^ but for clarity (see eqs 1 and 2), *i* is the experimental current density, σ is the conductivity, ϕ is the overpotential *E*–*E*_0_ (i.e., *E*_0_ is taken as the zero current point from the voltammogram), *i*_0_ is the exchange current density, *n* is the number of electrons, *F* is the Faraday constant, α is the dimensionless charge-transfer coefficient, *R* is the universal gas constant, and *T* is the absolute temperature.^56^
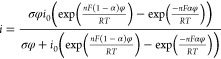
1
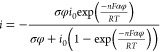
2

Equation 1 describes the anodic scan direction (dissolution), whereas eq 2 describes the cathodic scan (deposition). However, as we typically observe a characteristic nucleation loop in the cyclic voltammogram (refer to Figure 1a), which is not considered in this relation, eq 2 is of limited use. Nonetheless, the *i*–*E* curve-fitting method using eq 1 only has three fit parameters, which are *i*_0_, *E*_0_, and σ. All other parameters are constants, but it is worth noting that the charge-transfer coefficient, α, is set equal to 0.5 in our calculations. The charge-transfer coefficient can be a variable, e.g., for electrolytes with a more pronounced inflection at *E*_0_ potential, but we do not observe this with the AlCl_3_:Acet-Cl electrolytes. Furthermore, allowing α to vary as a fit parameter had a negligible effect on the resulting fits. A traditional objective minimization function (see eq 3) is used to fit the data via the chi-squared method.^57,58^
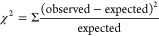
3

We typically find χ^2^ values on the order of 10^–3^, which show the goodness of fit between theory and experiment. As can be seen in Figure 1a, we find good agreement between the experimental CV trace and the predicted current based on eq 1 over the 900 mV potential fitting range. As can be seen in Table 1, we estimate a conductivity of 12.9 ± 1.4 mS cm^–1^, and we find good agreement in the predicted *i*_0_ and *E*_0_ fitted values to experimental data in Figure 1a. A simpler linear regression analysis to the voltammetric data can also be used. Specifically, by taking the best fit line to this current–potential data (0.00168 A V^–1^) and considering the separation distance (1.5 cm) between the working and counter electrodes, in addition to the working electrode surface area (0.196 cm^2^), we calculate a conductivity of 12.8 mS cm^–1^. This simply highlights that our modified Butler–Volmer formalism is estimating values expected from a rudimentary specific conductivity calculation. For reference, our previous work reported a conductivity of 9.7 mS cm^–1^, but this was for an AlCl_3_:Acet-Cl 1.5:1 mole ratio formulation.^35^ The larger conductivity value in the present study is attributed to the higher concentration of Al species resulting from the 2:1 mole ratio (discussed in more detail below). We postulate that the amidine-based ILA used here has a relatively high conductivity (compared to liquids made from similar LB salts, e.g., urea) as a result of the increased Lewis acidity that provides a higher concentration of charge carriers, in addition to the fact that it is an ionic Lewis base salt. Recent work by Schoetz et al. has utilized this method to extract conductivity values from imidazolium-based liquids.^59^ One shortcoming of the method we discuss is that it may only be applicable to concentrated electrolyte solutions typified by the AlCl_3_:Acet-Cl formulations demonstrated here for which ion concentrations are ca. 7 M. As such, these do not typically yield diffusion-limited voltammetric responses. In contrast, work in dilute aqueous media generally show peaked voltammetric responses governed by diffusion. Therefore, our methodology is not applicable to the latter situation. We are only in the early stages of developing this theory, and the limitations will become clearer as we explore more diverse chemical systems. Nonetheless, the methodology presented here represents a novel approach to extract conductivity from voltammetric data, and in the next section, we will compare it to more traditional measurement methods.

**Table 1:** All *i*–*E* Curve-Fitting Parameters^a^

parameter	value
fit parameters	
σ (mS cm^–1^)	12.9 ± 1.4
*i*_0_ (mA cm^–2^)	4.7 ± 2.2
*E*_0_ (V)	–0.06 ± 0.02
constants	
Α	0.5
*n*	3
*F* (C mol^–1^)	96,485
*R* (J mol^–1^ K^–1^)	8.314
*T* (K)	296

a The top three show the fitting (variable) parameters, whereas the bottom shows the five constant values.

### Impedance-Based Conductivity Measurements

A common way to estimate the ionic conductivity of electrolyte solutions is through AC impedance measurements. Broadband EIS data are often collected and fitted to an electrochemical equivalent circuit to find the ohmic resistance.^42,46,60^ The resistance can be used to calculate the conductivity in a previously calibrated cell using a cell constant, which is specific to the geometrical arrangement of the measurement electrodes and exposed surface area. As detailed in the Experimental Section and in ESI 2, a bespoke jacketed glass cell and PTFE cap were calibrated against a wide range of seven different conductivity standard solutions. We find a cell constant of *K* = 0.377 ± 0.002 cm^–1^, which is used to calculate conductivity values from similar measurements with our ILA electrolytes.

**Figure 2:**
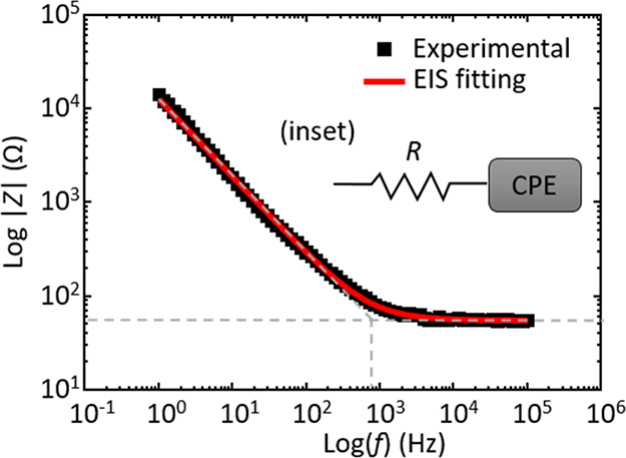
Experimental impedance modulus plot (filled black squares) and corresponding fit (solid red line) to an R-CPE electrochemical equivalent circuit (see the inset) for the AlCl_3_:Acet-Cl 2:1 formulation. The dashed gray lines are overlaid as a visual aid for estimating the capacitance and resistance. Collected at 0 V with a perturbation amplitude of 10 mV.

An exemplar Bode plot showing the impedance (*Z*) modulus from a broadband EIS spectrum is presented in Figure 2, which was collected at 0 V over the frequency (*f*) range from 100,000 to 10 Hz for the AlCl_3_:Acet-Cl 2:1 formulation. The filled black squares are the experimental data, and the solid red line is the fit to an electrochemical equivalent circuit (*R*-CPE; see the inset of Figure 2). From the plot, we can estimate the capacitance (*C*) and resistance (*R*) values. At high frequencies (>1 kHz), the resistive element dominates, and the plateau with respect to the *y*-axis in the high-frequency region suggests an *R* value of ca. 50 Ω. Alternatively, at low frequencies (<1 kHz), the capacitive element dominates. By approximating the crossover frequency point between the high and low regions on the *x*-axis (using the dashed gray lines in Figure 2), we estimate a frequency of ca. 750 Hz, which corresponds to the maximum frequency in an admittance plot. By a simple estimation of *C* = −1/(2·π·*f*·*Z*), we calculate a capacitance of ca. 20 μF cm^–2^. More formally, the data are fitted to an *R*-CPE electrochemical equivalent circuit using a χ^2^-based minimization objective function via the complex nonlinear least squares method. As can be seen, we find good agreement between the experimental and predicted data over the entire frequency range, and we typically find χ^2^ values on the order of 10^–3^. The fit estimates a *C* value of 17 ± 4 μF cm^–2^ and an *R* value of 53.9 ± 4.9 Ω, which confirm our visual estimations. Finally, by using this *R* value in conjunction with the cell constant from the calibrated cell, we calculate a conductivity of 7.0 ± 0.6 mS cm^–1^ for the AlCl_3_:Acet-Cl 2:1 formulation from the EIS-based method. It is worth noting that even if we only examine the low-frequency portion of the EIS spectrum, the estimated resistance values are similar to values when the entire broadband EIS spectrum is fit.

For comparative purposes, a commercial conductivity probe returns a conductivity value of 5.8 ± 0.2 mS cm^–1^ for the AlCl_3_:Acet-Cl 2:1 formulation. Interestingly, there appear to be differences in the estimated conductivity based on the measurement technique. The commercial conductivity probe is likely using an (undisclosed) single-frequency impedance measurement, whereas a broadband EIS measurement collects data over a wide range of frequencies. This difference could have an impact on the conductivity estimates, but it is likely to be relatively small as they are both based on an AC impedance measurement. The voltammetric method does suggest a higher conductivity value, by a factor of two, compared to the impedance-based methods, but importantly, they are all of the same order of magnitude. This provides some support to the underlying model we have outlined in the previous section. These observed variances in outcome between techniques may originate either in the differences of the surface condition of the measurement electrode or perhaps from the relative time scales over which the measurements are made. The translational modes of the dominant current carrying species may vary according to frequency. In the following sections, we will examine several different measurement parameters to test the voltammetric-based conductivity measurement method.

### Potential Scan Rate Effects

**Figure 3:**
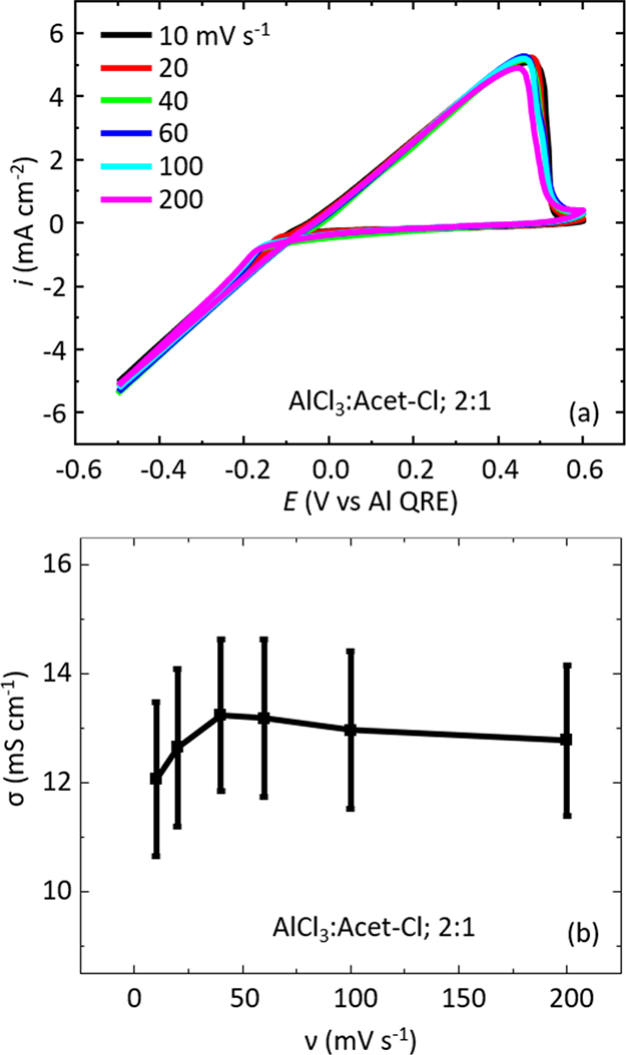
(a) Overlay of cyclic voltammograms at five potential scan rates for the AlCl_3_:Acet-Cl 2:1 formulation and (b) plot of conductivity (from *i*–*E* curve fitting) as a function of scan rate. Error bars represent the standard deviation of 3 replicate measurements.

The voltammetric response was next examined across a range of potential scan rates (ν). Figure 3a shows representative cyclic voltammograms for the AlCl_3_:Acet-Cl 2:1 formulation at 10, 20, 40, 60, 100, and 200 mV s^–1^ scan rates. Interestingly, we find very minimal scan rate dependence for the Al deposition/dissolution redox process. The onset potential for Al deposition occurs at −0.15 V, and the Al dissolution current peaks at 5 mA cm^–2^ at +0.5 V for all curves. The characteristic, symmetrical current–potential response is retained for all scan rates, and this includes the linear region from −0.5 to +0.4 V in the anodic scan direction that is fit to eq 1 to estimate the conductivity. The data in Figure 3b show the calculated conductivity as a function of scan rate. The conductivity values are independent of the scan rate at 13 mS cm^–1^ (within experimental uncertainty). Therefore, unless stated otherwise, the 100 mV s^–1^ scan rate was used for testing. The lack of any observed scan rate dependence here suggests that there is no mass-transport limitation. This may be due to the very high concentration of reducible Al^3+^ species (ca. 7 M) present and may also reflect a dynamic balance between the depletion of Al^3+^ in the diffusion layer and the rapid growth of the Al metal phase on the electrode surface.

### Width of Potential Excursion

**Figure 4:**
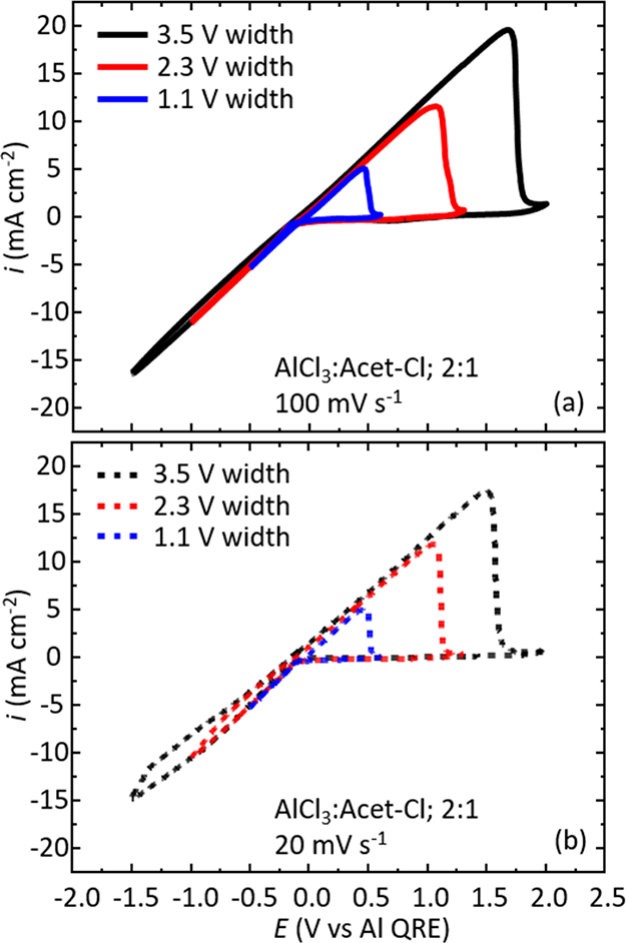
Overlay of cyclic voltammograms for the AlCl_3_:Acet-Cl 2:1 formulation at 1.1, 2.3, and 3.5 V potential widths collected at (a) 100 and (b) 20 mV s^–1^.

Another variable to consider is the width of potential excursion for the Al deposition/dissolution redox response (i.e., the absolute values of the anodic and cathodic limits). These limits are important as they dictate which processes occur (thermodynamics) and the rate (kinetics) at which they take place. The width of the potential window in which the deposition/dissolution process is stable and repeatable also impacts directly the operational specifications for any battery system containing such an electrolyte. For example, it would determine the maximum voltage limits applicable during charging. Cyclic voltammograms of the AlCl_3_:Acet-Cl 2:1 electrolyte were recorded using three different potential excursion values and two potential scan rates, Figure 4. Here, the solid line traces correspond to 100 mV s^–1^ CV data (Figure 4a), and the dashed line traces represent 20 mV s^–1^ CV data (Figure 4b). As we have previously mentioned, for the 1.1 V potential width, we see only negligible differences in the voltammetric signal between the two scan rates. This trend is also observed for the 2.3 V potential width. The 3.5 V potential width, however, does show some small differences with the slower 20 mV s^–1^ scan rate. Specifically, a slightly decreased peak dissolution current is observed, and the cathodic potential region shows signs of deviation from the linear response.

Interestingly, for the 100 mV s^–1^ traces, the cyclic voltammograms effectively build on top of each other as the potential width is increased. The *i*–*E* gradients in the anodic sweep remain identical, displaying the characteristic linear response, which yield similar (within error) conductivity values (12.9 ± 1.4, 12.5 ± 1.4, and 12.0 ± 1.6 mS cm^–1^ for the 1.1, 2.3, and 3.5 V potential widths, respectively). The linear response is largely retained at the 20 mV s^–1^ scan rate for the 2.3 and 3.5 V potential widths, but they do show some minor deviations. As a result, this does have a quantifiable impact on the estimated conductivity values, i.e., 12.6 ± 1.4, 11.5 ± 1.4, and 10.8 ± 1.5 mS cm^–1^ for the 1.1, 2.3, and 3.5 V potential widths, respectively. It is interesting that we are able to push the limits of the *i*–*E* curve-fitting method to fit data over nearly a 3 volt potential range. Importantly, the change in potential excursion does not appear to alter the underlying (electro)chemistry we are observing but merely the extent to which it occurs.

### Voltammetric Cycling Data

**Figure 5:**
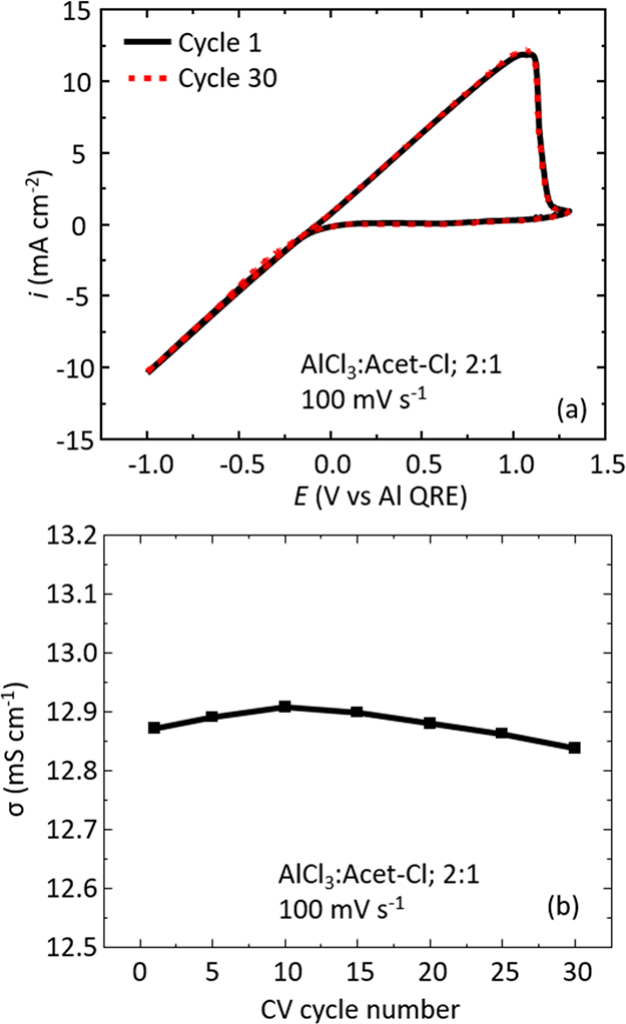
(a) Overlay of CV cycle 1 (solid black line) and cycle 30 (dashed red line) for the AlCl_3_:Acet-Cl 2:1 formulation at a scan rate of 100 mV s^–1^. (b) Corresponding conductivity data estimated from *i*–*E* curve fitting as a function of CV cycle number.

The CV cycle number was also examined to see if there was any effect. Data in Figure 5a show that there are no discernible differences from the 1st cycle (solid black trace) to the 30th cycle (dashed red trace) in the CV response associated with Al deposition/dissolution. The curve shape remains identical (i.e., the correlation coefficient between the two data sets is *R*^2^ = 0.999). The *i*–*E* curve-fitting method was used to extract the conductivity, and we find over the 30 cycles that the conductivity remains constant at 12.9 mS cm^–1^ (see Figure 5b). While this is not the focus of the present study, it presages well for future practical applications where this electrolyte may experience 100s to 1000s of cycles.

Lastly, it is worth commenting here on our reference electrode. It is accepted practice in the community to use a quasi-reference electrode that consists of a bare metal wire in non-aqueous RTIL/ILA/DES liquids. In Sections 3.4 to 3.6, we provide evidence throughout all the CV data that the Al QRE is stable across multiple (repeat) measurements that have been carried out over months of use. The position of the *i*–*E* gradient on the potential axis does not impact the conductivity estimated from our method, but rather it is the magnitude of the gradient itself that is the principal factor in influencing the conductivity value.

### *iR* Compensation Effects

**Figure 6:**
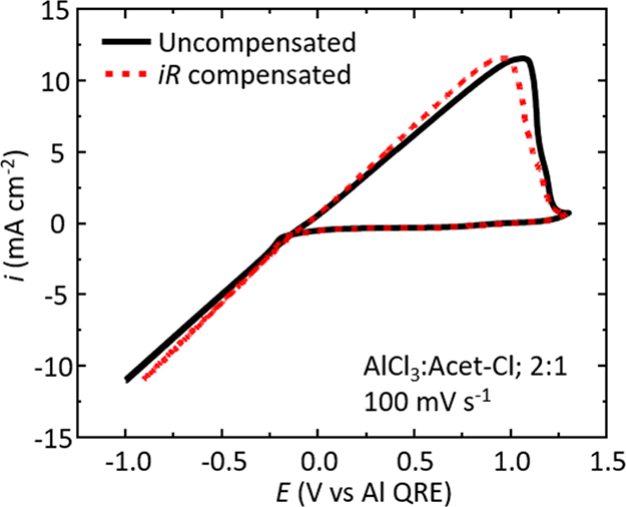
Overlay of cyclic voltammograms with uncompensated (solid black trace) and *iR*-compensated (dashed red trace) data for the AlCl_3_:Acet-Cl 2:1 liquid at a scan rate of 100 mV s^–1^.

The characteristic linear current–potential behavior was examined to see if *iR* effects were influencing the data. Cyclic voltammograms were corrected for ohmic drop by using the measured resistance for the AlCl_3_:Acet-Cl 2:1 liquid (53.9 ± 4.9 Ω). Figure 6 overlays an *iR* uncompensated cyclic voltammogram (solid black trace) and an *iR*-compensated cyclic voltammogram (dashed red trace) at a scan rate of 100 mV s^–1^. The *iR*-compensated cyclic voltammogram shows a very similar response to the uncompensated cyclic voltammogram, but the current–potential gradient (from −1 V to ca. +1 V) is slightly increased. We estimate conductivities of 13.0 and 14.3 mS cm^–1^ for the uncompensated and *iR*-compensated data, respectively. While this is a quantifiable difference, the overall observation is not sufficient to suggest that the *iR* effects are giving rise to the characteristic linear current–potential behavior.

For this formulation, AlCl_3_:Acet-Cl 2:1, we do not observe a peaked response for the electrochemical deposition of Al under the studied parameters. Therefore, under these experimental conditions, our data suggest that the Al deposition response is not limited by mass transport and diffusion of the electroactive species to/from the electrode surface.

### Temperature-Dependent Conductivity

**Figure 7:**
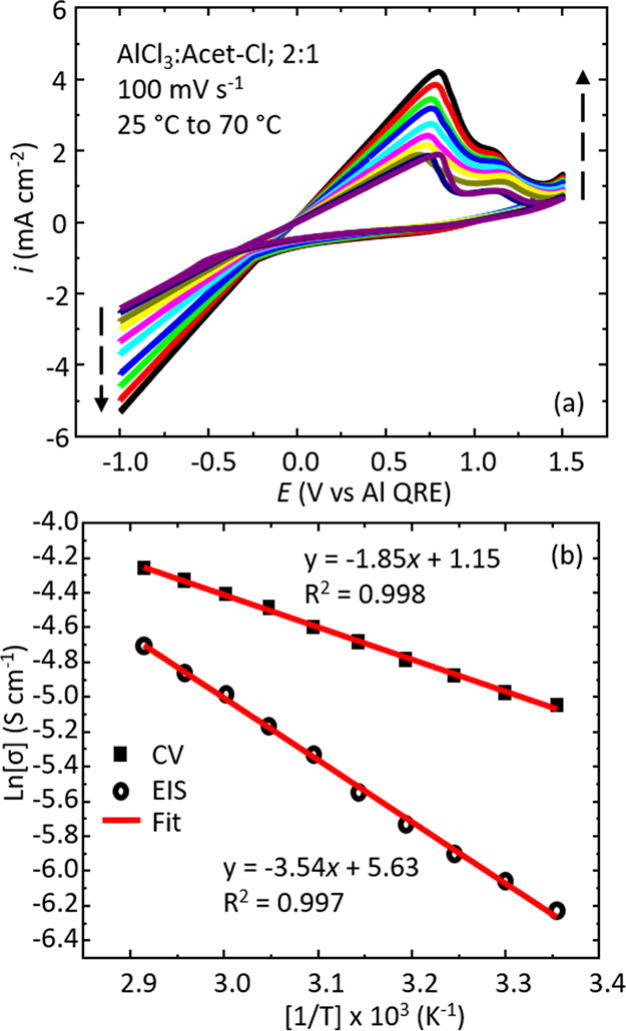
(a) Overlay of cyclic voltammograms measured from 25 to 70 °C at 100 mV s^–1^. The dashed black arrows point toward increasing temperatures. (b) Arrhenius plots of temperature-dependent conductivity data from EIS data (empty black circles) and CV data (filled black squares). The best fit line is shown for the two data sets along with the line equation and *R*^2^ coefficient.

The temperature-dependent behavior of the AlCl_3_:Acet-Cl 2:1 electrolyte was examined in a jacketed glass cell. The temperature was varied from 25 to 70 °C. The cyclic voltammograms in Figure 7a show similar behavior to what we have reported in previous sections, where a characteristic linear response is observed over a potential range in excess of 1.5 volts. As the temperature increases, the current response (and subsequently the *i*–*E* gradient, which informs on the conductivity) increases. One noticeable difference is the appearance of a second, smaller anodic oxidation peak around +1.2 V that follows the larger main Al dissolution peak. The additional dissolution peak could be a consequence from the formation of different morphological Al deposits (e.g., nanodeposits versus bulk metal deposits)^61,62^ or result from an alloy formed between Al and the Pt working electrode.^55^ For the purposes of the discussion here, however, the second dissolution peak assignment is out of the scope of our work.

The energy of activation (*E*_a_) can be estimated from temperature-dependent data. Figure 7b shows an Arrhenius plot of temperature-dependent conductivity data measured from the CV method and conductivity measured from EIS data. We find activation energies of 15 kJ mol^–1^ for CV data and 30 kJ mol^–1^ from EIS data. The difference in *E*_a_ values suggests that we may be probing different phenomena (on different timescales) between the two conductivity measurements. Nonetheless, these activation energies are consistent with previous reports for similar ILAs demonstrating Arrhenius behavior.^17,29,35,42,44,63,64^ Overall, we have demonstrated that the voltammetric-based conductivity measurement can be performed across a range of different measurement parameters.

### Effect of the Lewis Acid:Base Ratio

**Figure 8:**
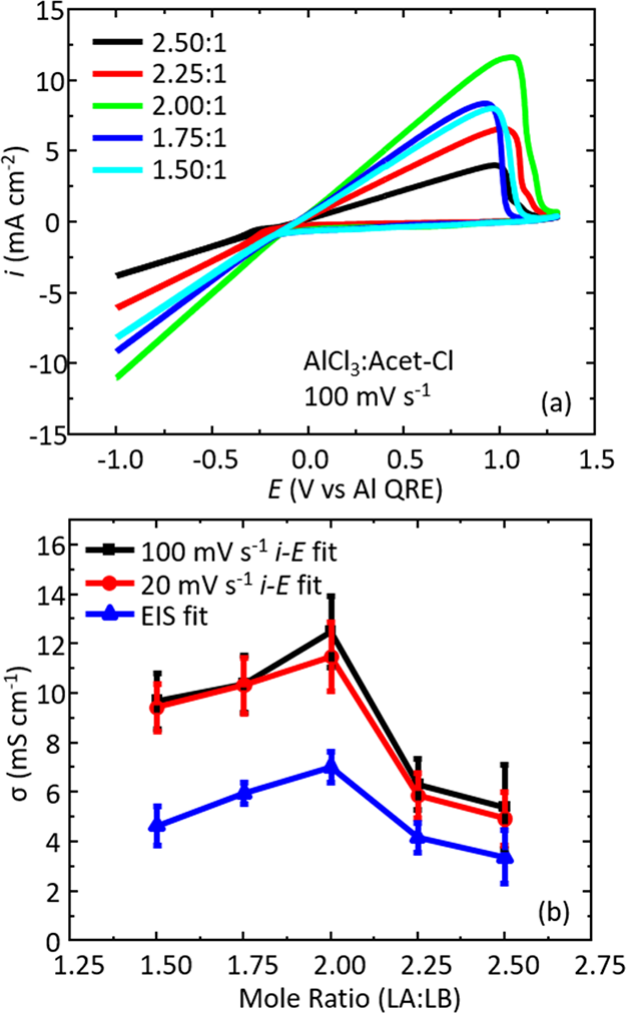
(a) Cyclic voltammograms at a scan rate of 100 mV s^–1^ for different mole ratios of LA:LB for the AlCl_3_:Acet-Cl system. (b) Overlays of the conductivity as a function of mole ratio for three different methods: 100 mV s^–1^*i*–*E* fitting (black squares), 20 mV s^–1^*i*–*E* fitting (red circles), and EIS fitting data (blue triangles). Error bars represent the standard deviation of 3 replicate measurements.

In the previous sections, the focus was the effect of physical measurement parameters. In this section, we shift the focus to chemical parameters, namely, the composition of the liquid. To examine the effect of the mole ratio of LA:LB, we have prepared a range of liquids for testing. Specifically, we find that a liquid is formed within the range LA:LB of 1.50:1 to 2.50:1. Outside this range, a stable liquid is not formed. Data in Figure 8a show the voltammetric behavior of the liquids as a function of mole ratio. They all show similar peak shapes, yet very different current magnitudes, but importantly, they all show the characteristic linear response suitable for *i*–*E* curve fitting. The 2.00:1 mole ratio shows the largest current signal and the highest *i*–*E* gradient. Moving higher or lower from this mole ratio, we find that the response decreases. A plot of the conductivity versus mole ratio is shown in Figure 8b, which highlights conductivity data measured from three methods: (i) *i*–*E* fitting at 100 mV s^–1^, (ii) *i*–*E* fitting at 20 mV s^–1^, and (iii) EIS fit data. Here, we see an increase in the conductivity from 1.50:1 to 2.00:1 followed by a decrease in conductivity toward higher mole ratios above 2.00:1. There are only minor differences between the voltammetric-based conductivity methods at the two different scan rates (as expected), and the EIS measured conductivity data trend mirrors that from the voltammetric method. The conductivities from the voltammetric-based method are a factor of two higher than those determined by the EIS-based method for all compositions studied. Earlier, we have speculated regarding the origins of these differences; however, the conductivity values are of the same order of magnitude and the compositional trends are the same, which provides validity to the underlying *i*–*E* curve-fitting model. It is worth noting that any changes in the, e.g., Lewis acidity, viscosity, and electrode surface area during the measurement are not significant to impact the bulk conductivity we are extracting from this method.^35^ Additionally, the small amount of impurities (1–2%; refer to Experimental Section) may impact the numerical value of conductivity for a liquid, but, as we demonstrate, it does not impact the validity of the *i*–*E* curve-fitting method we outline.

The majority of reports with similar chloroaluminate electrolytes find a related trend in conductivity as a function of mole ratio,^17,29,31,44,64^ where there is an optimal mole ratio to maximize conductivity for a particular electrolyte, yet some reports indicate that this trend may not always be the case.^14,25,45^ To the best of our knowledge, formulations of chloroaluminate liquids >2.00:1 are not common. Using our synthetic method, we have been able to generate liquids across a wide compositional range. These are highly Lewis acidic formulations with concentrations of [Al] ranging from 6.7 to 7.7 mol dm^–3^ for AlCl_3_:Acet-Cl 1.50:1 and 2.50:1, respectively (for reference, the AlCl_3_:EMIM-Cl 1.50:1 liquid has a [Al] of 5.7 mol dm^–3^). It is worth noting, however, after sitting overnight at room temperature that the AlCl_3_:Acet-Cl 2.50:1 formulation does solidify into small grainy/crystalline solids. This suggests that we could be making a supersaturated solution that eventually crystallizes upon cooling/sitting but could also indicate the formation of higher-order polymeric anions of the form [Al_*x*_Cl_3*x*+1_]^−^ as noted previously.^25,65^ The solution can be re-heated/stirred, but the electrochemical performance is significantly diminished when retested. We do not find this behavior for the AlCl_3_:Acet-Cl 2:1 formulation.

Lastly, we have also included viscosity data in ESI 3, Figure S3, for the mole ratio study. Here, we can see that the lowest viscosity is achieved from the AlCl_3_:Acet-Cl 2:1 electrolyte with a value of 37 ± 4 cP. At lower and higher mole ratios than 2:1 (LA:LB), we find that the viscosity increases and the error (standard deviation) in the measurement increases as well. Nonetheless, the viscosity data are in agreement with the conductivity data, which show an inverse relationship with one another.

### Applicability of the *i*–*E* Fitting Method with Various ILAs

In the final section, we have prepared a range of different liquids for testing against our voltammetric conductivity measurement method. Table 2 shows data for four different ILAs, i.e., two AlCl_3_:Acet-Cl formulations, AlCl_3_:acetamide, and AlCl_3_:urea, alongside a more conventional IL electrolyte AlCl_3_:EMIM-Cl. The Lewis acid:base mole ratio is listed for comparative purposes along with conductivity data measured from the voltammetric-based method (potential scan rate of 100 mV s^–1^), broadband EIS, and a commercial probe. Literature values are also provided with the corresponding measurement temperature. The CV data are shown in ESI 4, Figure S4, and demonstrates the characteristic linear *i*–*E* response suitable for data fitting for all liquids.

**Table 2:** Tabulated Data for Several Different ILA Electrolytes Providing the Mole Ratio (LA:LB) and Conductivity (σ) as Measured from Voltammetric Fitting, Broadband EIS, and from a Commercial Conductivity Probe^a^

electrolyte	mole ratio LA:LB	σ/mS cm^–1^ voltammetric fitting (23 ± 3 °C)	σ/mS cm^–1^ broadband EIS (27 ± 2 °C)	σ/mS cm^–1^ commercial probe (25 ± 2 °C)	σ/mS cm^–1^ literature [ref.]
AlCl_3_:Acet-Cl	2.0:1	12.5 ± 1.4	7.02 ± 0.63	5.8 ± 0.2	
AlCl_3_:Acet-Cl	1.5:1	9.7 ± 1.1	4.65 ± 0.77	4.0 ± 0.4	9.7 (18 °C)^35^
AlCl_3_:EMIM-Cl	1.5:1	19.7	10.7	13.7	17 (25 °C)^45^
AlCl_3_:acetamide	1.5:1	2.8	1.5	1.2	2.5 (40 °C)^31^
AlCl_3_:urea	1.5:1	0.77	0.63	0.49	1.15 (25 °C)^25^

a Literature values are also provided.

Interestingly, there are differences in the conductivity measured between the techniques for the same liquid, which indicates that to some extent, the value is dependent on the method. However, the difference is less pronounced when comparing the EIS and commercial probe data. This is probably because these two techniques are based on the same impedance measurement principle. Importantly, the voltammetric-based conductivity measurement method does estimate reasonable conductivity values, even when compared to literature values. We may in fact expect some differences in the conductivity values measured in our work compared to literature values simply as a result of the different synthetic methods employed. Our liquids are not prepared under rigorous inert atmospheric conditions (such as provided with a glovebox), and we utilize heating during the reaction. There may also be some minor variability between our work and previously published data due to different purities of the salts used and different measurement temperatures. Nonetheless, our novel voltammetric-based conductivity measurement method provides a reasonable estimation of ionic conductivity for chloroaluminate electrolytes without the need for an additional measurement or sensor. Lastly, this work has highlighted the significant improvement in conductivity achieved with the AlCl_3_:Acet-Cl 2:1 formulation, which is a substantial improvement in electrolyte development for Al-ion battery materials.

## Conclusions

In this work, we have demonstrated a novel, simple, and effective method for extracting the conductivity of ILA electrolytes based on fitting the characteristic linear portion of the voltammogram. The new method is benchmarked to data from both broadband impedance-based measurements and that from a commercial conductivity probe. The data derived from voltammetric curve fitting gives values of conductivity that are close to a factor of two larger than the impedance-based measurements but are nevertheless in close agreement with other literature values. This may be the result of differences in the response times of molecular dynamics imposed by DC and AC methods. The measured values of conductivity for all methods are of the same order of magnitude, and the new method predicts similar conductivity trends with respect to compositional data. We observe no dependence on the potential scanning rate and CV cycle number. The absolute values of the cathodic and anodic potential limits examined and uncompensated *iR* effects only have a minor influence on the estimated conductivity. Arrhenius behavior is observed with temperature-dependent conductivity data, and we find activation energies comparable to literature sources. We also show that the voltammetric-based method is applicable to measure the conductivity of a range of other ILAs. Another significant result from this work is the high conductivity obtained with the AlCl_3_:Acet-Cl 2:1 formulation at ca. 7 mS cm^–1^. This represents a substantial improvement over conductivities from urea- and acetamide-based electrolytes. Lastly, it is important to reiterate that the amidine-based salts are a much more cost-effective option compared to current state-of-the-art imidazolium-based salts, which is important when considering scale-up of these electrolytes for Al-ion battery applications. Future work will examine the time-dependent rheological performance of the AlCl_3_:Acet-Cl 2:1 electrolyte and its electroplating characteristics.

## Data Availability

Data will be made available on request.

## Supporting Information Available

Electrochemical cells, EIS conductivity cell calibration, mole ratio-dependent viscosity data, and demonstration of the *i*–*E* fitting method for different ILAs (PDF)

## Notes

A.J.L.: conceptualization, methodology, validation, formal analysis, investigation, writing–original draft, writing–review and editing, visualization, supervision, and project administration. I.S.: methodology, validation, formal analysis, investigation, and visualization. E.B.: methodology, validation, formal analysis, investigation, and visualization. I.E.: conceptualization, methodology, formal analysis, and writing–review and editing. S.V.: conceptualization, methodology, and formal analysis. A.R.H.: conceptualization, methodology, resources, writing–review and editing, supervision, project administration and funding acquisition. C.J.Z.: conceptualization, methodology, and formal analysis. K.S.R.: conceptualization, methodology, resources, writing–review and editing, supervision, project administration, and funding acquisition. I.S. and E.B. contributed equally.
