# Supplementary material for: Measuring and Enhancing the Ionic Conductivity of Chloroaluminate Electrolytes for Al-Ion Batteries
Source: J Phys Chem C Nanomater Interfaces. 2023 Jul 6;127(28):13866–76. doi: 10.1021/acs.jpcc.3c02302 (PMC10364082; doi:10.1021/acs.jpcc.3c02302)
Supplement: Supplementary file 1 — jp3c02302_si_001.pdf [file jp3c02302_si_001.pdf]

Electronic Supporting Information (ESI)

Measuring and Enhancing the Ionic Conductivity of Chloroaluminate Electrolytes for Al-Ion Batteries

Anthony J. Lucio,[*,a] Iwan Sumarlan,[ǀ,a,b] Elena Bulmer, [ǀ,a] Igor Efimov,[c] Stephen Viles,[a] A. Robert Hillman,[a] Christopher J. Zaleski,[d] and Karl S. Ryder[a]

[a] Centre for Sustainable Materials Processing, School of Chemistry, University of Leicester, LE1 7RH, Leicester, U.K.

[b] Department of Chemistry, University of Mataram, Jl. Majapahit. No. 62, Mataram, Lombok, Indonesia

[c] Department of Chemical and Biological Engineering, University of Sheffield, Sheffield, S1 3JD, U.K.

[d] Biotechnology Group, School of Chemistry, University of Leicester, Leicester, LE1 7RH, U.K.

*Corresponding author: anthonyluciophd@outlook.com (A. J. Lucio)

[ǀ]These authors contributed equally

Table of Contents:

ESI 1. Electrochemical cells

ESI 2. EIS conductivity cell calibration

ESI 3. Mole ratio dependent viscosity data

ESI 4. Demonstration of *i*-*E* fitting method for different ILAs

ESI 5. SI References

ESI 1. Electrochemical cells

The two electrochemical cells used are schematically shown in **Figure S1**. The simultaneous CV/QCM measurements were done in a purpose-built cell, made from PEEK material, to physically fit the QCM resonator electrodes (**Figure S1a-b**); **(a)** is the main cylinder and **(b)** is the bottom cap. The total volume inside of the PEEK cylinder is 20 mL. Standard o-rings were used to prevent any liquid leaks and protect the QCM resonator electrode.

The EIS measurements were performed in a jacketed glass cell (**Figure S1c**). The total volume inside of the jacketed cell is 70 mL. The schematic shows the two different liquid layers with the ILA electrolyte in a brown colour on the bottom and the less dense viscous paraffin oil layer on top in a grey colour. The two cylindrical wires represent the Al electrodes used in the broadband EIS measurements. The paraffin oil layer is not a conducting liquid, so its presence does not impact on the measured data.

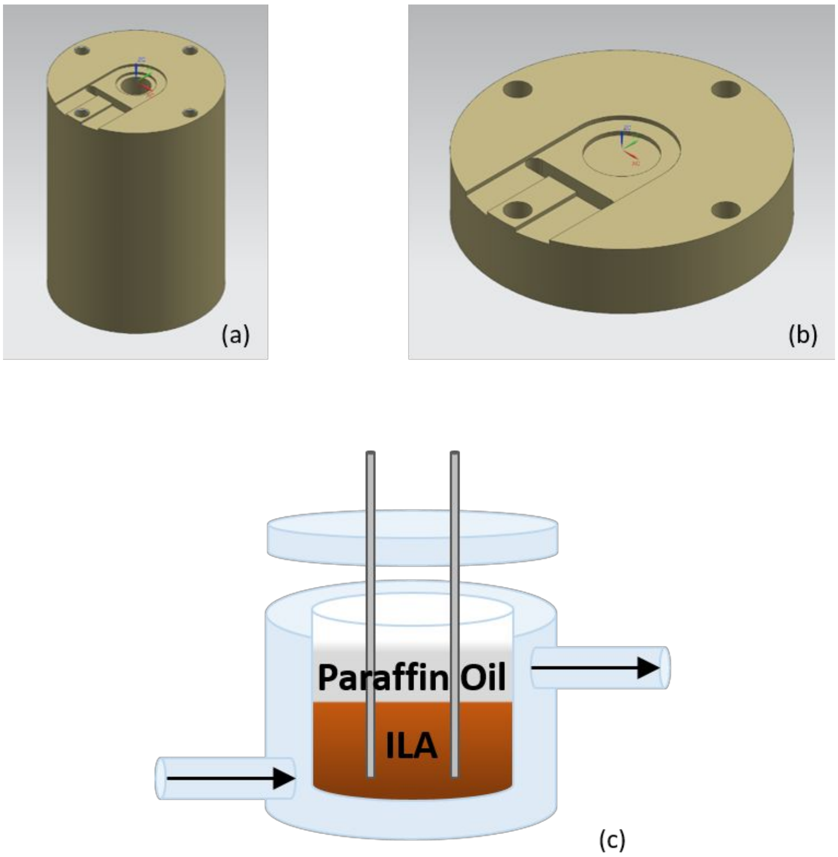

Figure S1. The purpose-built CV/QCM cell is shown in (a) main cylinder body and (b) bottom cap. These are screwed together with an o-ring. (c) A homemade jacketed glass cell for the broadband EIS measurements. The ILA electrolyte and paraffin oil layers are labelled with brown and grey colours, respectively. Two dark grey coloured wires represent the Al electrodes. The solid black arrows indicate the heated water flow for temperature-dependent measurements.

ESI 2. EIS conductivity cell calibration

The ionic conductivity of a solution can be measured from broadband EIS data. Here we have used seven different conductivity standard solutions (i.e., 44,479, 11,419, 8,863, 1,249, 885, 442, and 74 µS cm^{-1}; at 19 °C) to calibrate our homemade jacketed glass cell. **Figure S2a** shows a Bode plot of the impedance modulus as a function of frequency for the conductivity standard solutions. The plateau region at higher frequencies (i.e., 10^4 to 10^5 Hz) gives a visual estimation of the resistance, and we see the resistance decreases with more conductive solutions (i.e., the dashed black arrow points towards higher conductivities) as expected. The resistance values are quantitatively extracted from fitting EIS data to an electrochemical equivalent circuit. Here the data fit very well to a simple *R*-CPE model (see colour matched solid lines in **Figure S2a**; inset shows equivalent circuit), which consists of a resistor (*R*) in series with a constant phase element (CPE) to represent a non-ideal capacitor. The cell constant (*K*; cm^{-1}), which is dependent on the geometrical arrangement of the electrodes and electrode surface area, was determined from resistance (*R*; Ω) measurements of known conductivity (σ; S cm^{-1}) standard solutions using the equation $\sigma = K/R$. Specifically, the gradient of a plot of σ versus 1/*R* will yield the cell constant. From this calibration in **Figure S2b** we find a cell constant $K = 0.377$ (± 0.002) cm^{-1} that can be used to measure conductivities of other electrolyte solutions.

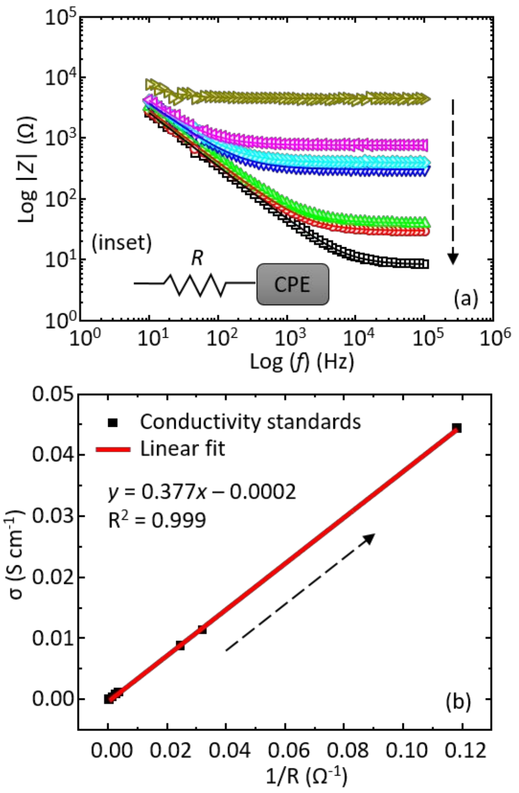

Figure S2. (a) Bode plot showing the impedance modulus versus frequency for the seven conductivity standards. The open symbols represent the raw data and the colour-matched solid lines represent the corresponding fit to an R-CPE electrochemical equivalent circuit (inset). (b) Calibration plot showing the conductivity versus the measured resistance values from the standard solutions. The best fit line is shown along with the line equation. The slope yields the cell constant (cm^{-1}). The dashed black arrows points towards increasing conductivity. Error bars (x-axis) are contained within the symbols.

ESI 3. Mole ratio dependent viscosity data

Electrolyte viscosity values were estimated from QCM data as shown previously.[1] In **Figure S3** we show the effect the mole ratio of LA:LB on the viscosity. The $AlCl_3$:Acet-Cl 2:1 liquid shows the lowest viscosity at 37 ± 4 cP. At mole ratios both below and above 2:1 (LA:LB) we can see the viscosity increases, and a sharper increase is observed at more Lewis acidic ratios. The error (standard deviation) in the measured data also increases as we move away from the 2:1 mole ratio. Overall, we find the 2:1 mole ratio to be the optimal composition from our tests.

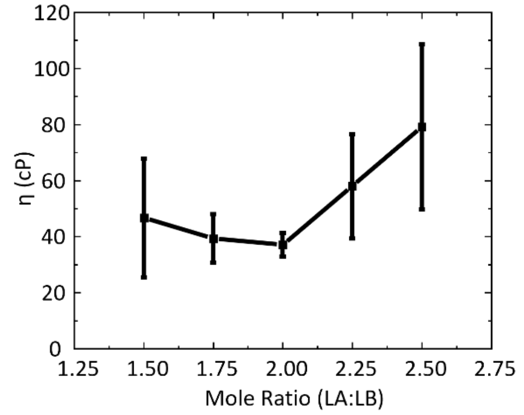

Figure S3. Mole ratio dependent viscosity data for the $AlCl_3$:Acet-Cl system.

ESI 4. Demonstration of *i-E* fitting method for different ILAs

The *i-E* curve fitting method was used to investigate several other ILA electrolytes prepared in a similar way. Data in **Figure S4** show overlaid experimental CV traces and corresponding *i-E* fits for (a) $AlCl_3$:EMIM-Cl, (b) $AlCl_3$:acetamide, and (c) $AlCl_3$:urea. All formulations use a 1.50:1 mole ratio. All CVs show linear *i-E* behaviour in the anodic scan with fitting operable over >1.5 volts. The $AlCl_3$:EMIM-Cl 1.50:1 liquid in **Figure S4a** shows similar behaviour to our $AlCl_3$:Acet-Cl 2:1 liquid but the higher currents yield a higher conductivity of 19.7 mS cm^{-1}. The $AlCl_3$:acetamide (**Figure S4b**) and $AlCl_3$:urea (**Figure S4c**) have significantly smaller estimated conductivities (and smaller current magnitudes) of 2.80 mS cm^{-1} and 0.77 mS cm^{-1}, respectively. Furthermore, the acetamide- and urea-based electrolyte CVs show signs of Al passivation in the anodic region near 1.0 V to 1.3 V as the current does not fall back to the baseline. This feature is not observed for the $AlCl_3$:EMIM-Cl 1.5:1 nor $AlCl_3$:Acet-Cl 2:1 liquids in our work but has been reported previously for other $AlCl_3$:EMIM-Cl liquids.[2, 3]

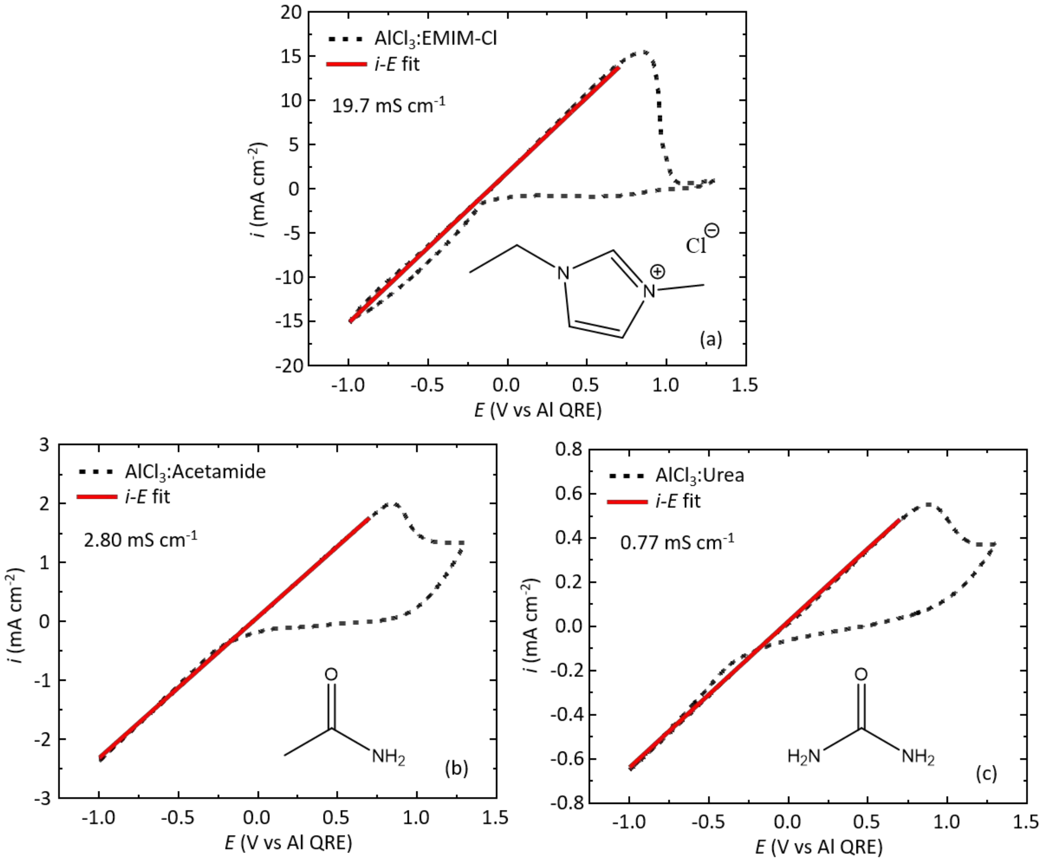

Figure S4. CVs showing a range of different ILAs: (a) $AlCl_3$:EMIM-Cl, (b) $AlCl_3$:acetamide, and (c) $AlCl_3$:urea. All formulations are using a 1.50:1 mole ratio. The scan rate is 100 mV s^{-1}. The dashed black lines are the experimental data and the solid red lines are the i-E curve fits. The calculated conductivity is also reported in each plot. The chemical structures of the Lewis base salt precursors are provided as insets.

ESI 5. SI References

(1) Lucio, A. J.; Efimov, I.; Efimov, O. N.; Zaleski, C. J.; Viles, S.; Ignatiuk, B. B.; Abbott, A. P.; Hillman, A. R.; Ryder, K. S. Amidine-based ionic liquid analogues with AlCl3: a credible new electrolyte for rechargeable Al batteries. *Chemical Communications* **2021**, *57* (77), 9834-9837, 10.1039/D1CC02680A. DOI: 10.1039/D1CC02680A.
(2) Böttcher, R.; Ispas, A.; Bund, A. Anodic dissolution of aluminum and anodic passivation in [EMIm]Cl-based ionic liquids. *Electrochemistry Communications* **2020**, *115*, 106720. DOI: https://doi.org/10.1016/j.elecom.2020.106720.
(3) Wang, C.; Creuziger, A.; Stafford, G.; Hussey, C. L. Anodic Dissolution of Aluminum in the Aluminum Chloride-1-Ethyl-3-methylimidazolium Chloride Ionic Liquid. *Journal of The Electrochemical Society* **2016**, *163* (14), H1186-H1194. DOI: 10.1149/2.1061614jes.
